# Cephalgien und Doppelbilder mit ungewöhnlicher Ursache

**DOI:** 10.1007/s00106-023-01402-x

**Published:** 2024-01-02

**Authors:** M. Götting, R. Zibell, M. Jungehülsing

**Affiliations:** Abteilung für HNO, Kopf- und Halschirurgie, Klinikum Ernst von Bergmann, Charlottenstraße 72, 14467 Potsdam, Deutschland

## Anamnese

**Abb. 1:**
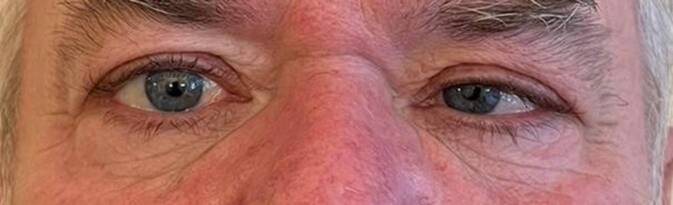
Spontane Adduktion des linken Auges beim Blick geradeaus

Ein 62-jähriger Patient berichtete bei seiner Vorstellung in unserer Rettungsstelle über seit einer Woche bestehende, rechts temporal betonte Cephalgien. Zudem sei es vor zwei Tagen plötzlich zu einem Einwärtsschielen des linken Auges gekommen. Seither bestünden nicht kreuzende Doppelbilder mit Zunahme beim Blick nach links (Abb. 1). Anamnestisch bestand kein Strabismus in der Kindheit. Fieber oder eine B‑Symptomatik wurden verneint. Als einzige Komorbidität gab der Patient eine medikamentös eingestellte arterielle Hypertonie an.

## Klinischer Befund und Diagnostik

**Abb. 2:**
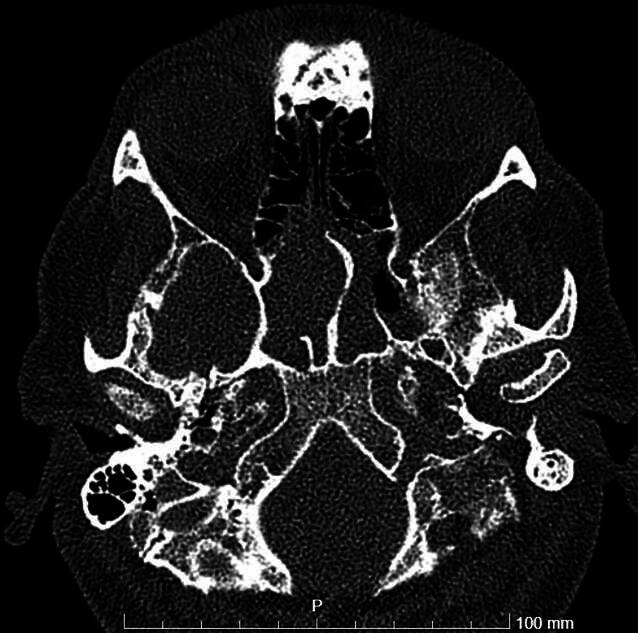
CT der Nasennebenhöhlen, transversale Schicht: *rechts* komplett, *links* teilverschatteter Sinus sphenoidalis

Eine zunächst neurologisch durchgeführte Untersuchung inklusive cranialer Computertomographie (cCT) und computertomographischer Angiographie (CTA) ergab keinen Hinweis für eine frische Ischämie, Blutung oder Raumforderung bei Totalverschattung der rechten Keilbeinhöhle sowie partieller Verschattung der linken Keilbeinhöhle (Abb. 2). Laborchemisch fielen allerdings erhöhte Infektwerte auf (Leukozyten 10,4 Gpt/l, CRP 52,4 mg/l), sodass eine craniale Magnetresonanztomographie (cMRT) mit Kontrastmittel durchgeführt wurde. Hier zeigte sich eine etwas inhomogene Kontrastierung des Sinus cavernosus links mit fraglichen punktuellen Kontrastmittelaussparungen ohne Nachweis einer Thrombose sowie die bereits in der cCT beschriebene Verschattung der Keilbeinhöhle. In der Liquordiagnostik zeigte sich mit einer unauffälligen Zellzahl kein Hinweis für eine sekundäre Meningitis. Hinweise für einen weiteren Infektfokus fanden sich weder in der Röntgenaufnahme des Thorax noch im Urinstatus.

Eine augenärztliche Vorstellung ergab beidseits einen normalen Visus sowie normalen Augeninnendruck bei eingeschränkter Abduktion des linken Auges (auch in Hebung und Senkung) und intakter Konvergenz. Doppelbildzunahme bei Blick nach links, Bielschowsky-Test negativ.

## Operative Therapie

**Abb. 3:**
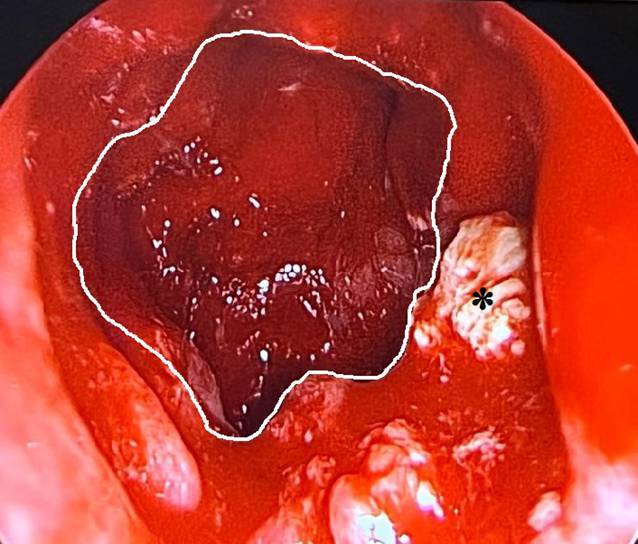
Endoskopisches Bild der eröffneten Keilbeinhöhle rechts (*weiße Umrandung*) mit aus der Keilbeinhöhle gewonnenem weißlichem Gewebe (*Stern*)

Es erfolgte eine endoskopische transseptale Sphenodotomie beidseits. Intraoperativ zeigten sich die Keilbeinhöhlen mit Pus sowie weißlich-bröckeligem Gewebe ausgefüllt (Abb. 3). Es wurden mikrobiologische Proben sowie Gewebe für die histologische Aufarbeitung gewonnen.

Wir begannen eine kalkulierte intravenöse antibiotische Therapie mit Ampicillin/Sulbactam (2000 mg/1000 mg) 3 × täglich.

## Wie lautet Ihre Diagnose?

**Diagnose:** Abduzensparese links durch akut exazerbierte chronische Mykose des Sinus sphenoidalis rechtsbetont

## Verlauf

Bereits am ersten postoperativen Tag zeigte sich die Abduzensparese regredient, was mit einer Verringerung der empfundenen Doppelbilder einherging.

Der Abstrich aus dem Sinus sphenoidalis ergab den Nachweis eines unkomplizierten Escherichia coli, sensibel gegen die begonnene antibiotische Therapie, sodass diese antibiogrammrecht für insgesamt 7 Tage fortgeführt werden konnte.

Der kulturelle Pilznachweis gelang nicht. Histologisch zeigte sich eine rezidivierende chronische, leicht pseudopolypös-hyperplastische Sinusitis ethmoidalis beidseits mit Sprosspilzkolonien nach Art von Aspergillus (Aspergillome) ohne Hinweis für Malignität.

Unter lokaler Pflege mittels Absaugen und NaCl-Nasenspülung gestaltete sich der weitere Verlauf unkompliziert. Der Patient konnte in deutlich gebesserten Allgemeinzustand am 4. postoperativen Tag in die ambulante Nachsorge entlassen werden.

**Abb. 4:**
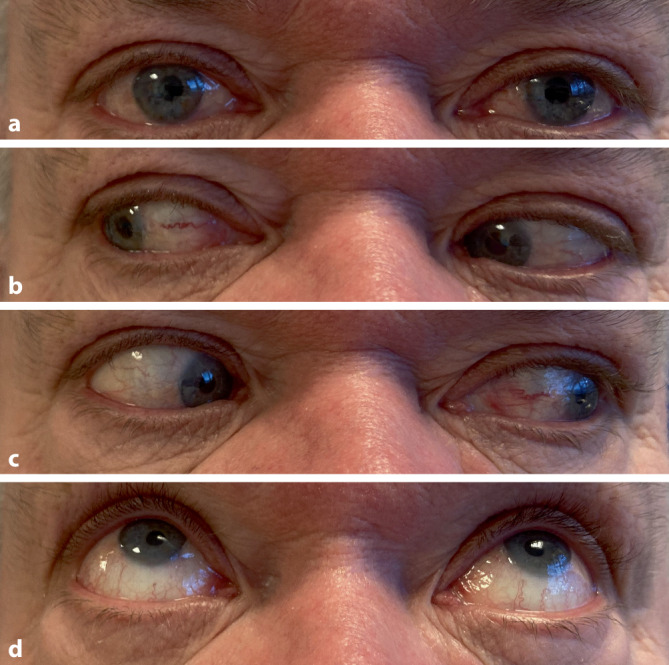
Einen Monat postoperativ *von oben nach unten*: Blick geradeaus, Blick nach rechts, Blick nach links, Blick nach oben: keine Residuen der vormaligen Abduzensparese nachweisbar

**Abb. 5:**
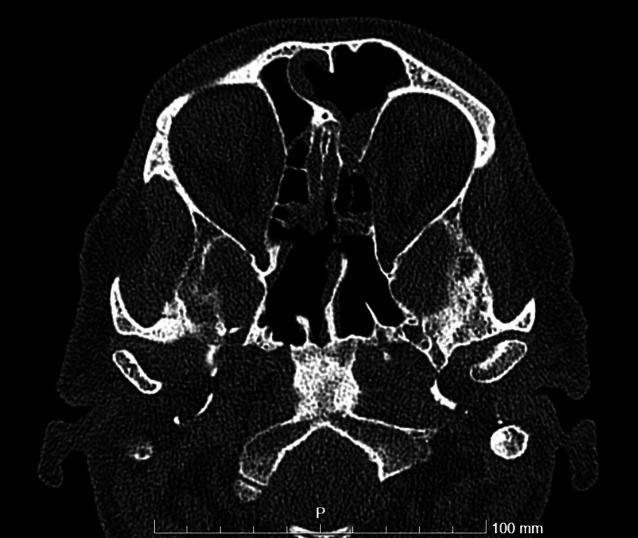
CT-NNH, transversale Schnittführung: postoperativ frei belüftete Sinus sphenoidales beidseits

In der klinischen Verlaufskontrolle einen Monat postoperativ zeigte sich eine komplette Restitutio ad integrum der Abduzensparese (Abb. 4) und in der durchgeführten CT-NNH ein frei belüfteter Sinus sphenoidalis (Abb. 5).

## Pathogenese und Differenzialdiagnose

Die Pathogenese der hier aufgetretenen N.-abducens-Parese und ihre möglichen differenzialdiagnostischen Ursachen erklären sich aus dem Verlauf des N. abducens.

Als VI. Hirnnerv liegt der Nucleus nervi abducentis in der Pons im Hirnstamm. Von hier läuft der N. abducens durch die Pyramidenbahn im Hirnstamm und durch den Sinus cavernosus. Schließlich erreicht er durch die Fissura orbitalis superior die Orbita. Hier innerviert er nur einen Muskel, den Musculus rectus lateralis, der für die Abduktion des Auges verantwortlich ist. Eine Parese führt zu einem Überwiegen des für die Adduktion verantwortlichen Musculus rectus medialis. Der betroffene Bulbus steht somit bei einer peripheren Abduzensparese nach innen [1].

Durch die enge Lagebeziehung der Sinus cavernosi beidseits der Sinus sphenoidalis ist eine Parese des N. abducens in dieser Region durch einen Prozess des Sinus sphenoidalis möglich.

Die ursächlichen Pathologien sind hierbei sehr vielfältig und reichen von Neoplasie über Mukozelen, Cholesteringranulome, Aspergillome, chronische Sinusitis sphenoidalis bis zu Hämangiomen [2].

Sofern möglich, ist die Therapie der Wahl die Sphenodotomie und hiermit Beseitigung des ursächlichen Prozesses. Zusätzlich kommen bei Bedarf antibiotische und antimykotische Medikamente zum Einsatz [3].

Weitaus häufiger als durch Prozesse im Sinus sphenoidalis werden periphere Abuzensparesen durch allgemeine neurologische Erkrankungen ausgelöst, meist treten die Paresen dann jedoch nicht isoliert auf.

Eine Abduzensparese in Kombination mit einer Okulomotoriusparese und Trochlearisparese sowie Hypästhesie im Bereich der Trigeminusäste V1 und V2 wird als Sinus-cavernosus-Syndrom bezeichnet. Die individuelle Ausprägung und Kombination der Hirnnervenschädigung kann jedoch je nach Pathologie sehr unterschiedlich sein, was sich auch in den möglichen Ursachen des Sinus-cavernosus-Syndroms widerspiegelt. Hier sind septische oder aseptische Sinus-cavernosus-Thrombosen, Hypophysenadenome, Aneurysma der Arteria carotis interna, Sarkoidose, Granulomatose mit Polyangiitis, Hämorrhagie infolge Trauma oder Apoplexie sowie das Tolosa-Hunt-Syndrom zu nennen [4].

Ferner können auch Tumoren und Entzündungen der Felsenbeinspitze (Gradenigo-Syndrom), Lyme-Borreliose, Nasopharynxtumoren, Schädelbasisfrakturen und erhöhter intrakranieller Druck für eine Abduzensparese verantwortlich sein [5].

## Fazit für die Praxis

- Prozesse im Sinus sphenoidalis können zu isolierten Abduzensparesen führen.
- Eine isolierte Abduzensparese bedarf einer weiteren Abklärung mittels Schnittbildgebung (bevorzugt MRT, gegebenenfalls CT).
- Eine isolierte, auch asymptomatische, Verschattung des Sinus sphenoidalis bedarf immer einer Therapie.
- Sollte eine medikamentöse Therapie fehlschlagen, ist eine frühzeitige chirurgische Intervention empfehlenswert.
